# Translation and cross-cultural adaptation of the Brazilian version of the Boston Residue and Clearance Scale (BR-BRACS)

**DOI:** 10.1590/2317-1782/e20240256en

**Published:** 2025-08-08

**Authors:** Giulia Beatriz Pozena Scaranelo, Suely Mayumi Motonaga Onofri, Leandro de Araújo Pernambuco, Roberta Gonçalves da Silva

**Affiliations:** 1 Laboratório de Disfagia e Programa de Pós-Graduação em Ciências da Saúde e Comunicação Humana do Departamento de Fonoaudiologia , Universidade Estadual Paulista "Júlio de Mesquita Filho" - UNESP - Marília (SP), Brasil.; 2 Departamento de Fonoaudiologia e Programa de Pós-Graduação em Saúde da Comunicação Humana da Universidade Federal de Pernambuco-UFPE, Recife/PE, Brasil.

**Keywords:** Translations, Cross-Cultural Comparison, Deglutition Disorders, Evaluation and Screening, Endoscopy

## Abstract

**Purpose:**

This study aimed to translate and cross-culturally adapt The Boston Residue and Clearance Scale (BR-BRACS) into Brazilian Portuguese and validate the image selection content for the scale.

**Methods:**

The project was approved by the Institution’s Ethics Committee under number 67715717.6.0000.5406. The process involved translating the scale into the target language, synthesizing the translations, and back-translating it into the original language. A literature review was conducted to define the concept of pharyngeal residue and ensure the content validity of the images representing the scale. Then, 50 fiberoptic endoscopic evaluations of swallowing were analyzed and submitted to three judges for visual-perceptual evaluation. Agreement among the judges was analyzed using Fleiss' Kappa test with a 95% confidence interval.

**Results:**

Discrepancies in lexical and syntactic contexts were identified during translation and resolved through a consensus among translators and authors. The back-translated versions were equivalent to the original. The images proposed for the scale showed near-perfect agreement among the judges.

**Conclusion:**

The translation, cross-cultural adaptation, and selection of images for visual-perceptual evaluation of pharyngeal residue in the BR-BRACS were completed with evidence of content validity.

## INTRODUCTION

Healthcare assessment, whether clinical or through various examinations, requires reliable procedures, protocols, and scales with diagnostic accuracy. Regarding the diagnosis of swallowing disorders, assessment instruments with scales that measure the degree of impairment are important for understanding the levels of dysfunction in swallowing biomechanics and analyzing the nature and prognosis of the condition at any point during patient care. These instruments, whether clinical evaluations or instrumental exams, are complementary and provide excellent feedback tools for families and patients. Additionally, they are part of a set of markers that assist professionals in clinical decision-making^(1,2)^.

Pharyngeal residues (PR) in oropharyngeal dysphagia are a risk marker for laryngotracheal aspiration. Although various scales exist for analyzing PR, they were predominantly published in American English and are mostly designed to classify PR in videofluoroscopic swallowing studies (VFSS)^(3-5)^. Only a few scales are available for analysis using fiberoptic endoscopic evaluation of swallowing (FEES)^(6-8)^. The Boston Residue and Clearance Scale (BRACS) is one of the scales available to enhance PR analysis via FEES – an excellent instrumental examination for evaluating this finding – and measure the levels of impairment associated with PR.

Translating and adapting PR classification scales into Brazilian Portuguese is essential to enhance diagnosis and clinical decision-making based on internationally recognized markers. Thus, this study aimed to translate and cross-culturally adapt the BR-BRACS into Brazilian Portuguese and validate the image selection content for the scale.

## METHODS

This study was approved by the Research Ethics Committee (CEP) under protocol number 67715717.6.0000.5406. To initiate the study, the primary author of the instrument was contacted via email and granted authorization for its use. The BRACS is an 11-point ordinal scale divided into 12 locations with four classifications of residue quantity (none/coating, mild, moderate, and severe). The identified residue locations are divided into two regions: Region 1 includes the lateral pharyngeal wall, posterior pharyngeal wall, base of tongue, valleculae, and tip of epiglottis; Region 2 includes the left lateral channel and left piriform recess, right lateral channel and right piriform recess, and the postcricoid region. The scale's scoring is based on the presence of residue in one or more locations and ultimately classifies swallowing efficiency based on stimulated or spontaneous swallows to clear the residue^(7)^.

BR-BRACS’ translation and cross-cultural adaptation followed the six-stage method by Beaton et al.^(9)^. This study phase carried out the first three stages – (1) Translation into the target language, Brazilian Portuguese; (2) Synthesis of the translations; and (3) Back-translation into the original language, American English, as described below:

### Stage 1 – Translation into the target language: Brazilian Portuguese

Two translators experienced in scientific healthcare translations, including other oropharyngeal dysphagia instruments, translated the scale from its original language (American English) into Brazilian Portuguese. The selection criteria for the translators were being native Brazilian speakers, fluent in the original language of the instrument, and experienced in healthcare translations. The translators had an average of 9 years of experience in the field and met all criteria. After being contacted, they were sent the file containing an instruction paragraph and the BRACS via email, with a 1-week deadline for translation. Each translator completed the translation independently, resulting in two versions (T1 and T2).

The instruction paragraph for using and scoring the BRACS was translated as part of the process. This paragraph was extracted from the original article and divided into 14 segments, sent to the translators for them to translate. After completing the translations, the paragraph and the scale proceeded to Stage 2.

### Stage 2 – Synthesis of the translations

The research committee, comprising the authors (with an average of 15 years of experience in the field of the translated instrument) and the two translators from Stage 1, held an online meeting to resolve lexical and syntactic discrepancies and create a consensus version (T1+T2) in Brazilian Portuguese. The meeting, held via Google Meet, was the first time the translators reviewed their translations together. Adjustments were made by consensus among the translators and the study authors, comparing the consensus version with the original text to identify the best equivalent terms commonly used in the specialty field in Brazilian Portuguese.

### Stage 3 – Back-translation into the original language (North American English) and cross-cultural adaptation

Two native English-speaking translators fluent in Brazilian Portuguese back-translated the Brazilian Portuguese consensus version (T1+T2) into the instrument’s original language (American English). After this process, any discrepancies in the back-translated version were discussed in a consensus meeting between the translators and researchers, resulting in the final version.

At the end of the stage, it was decided to compare the consensus version with the original version based on the translators’ and study authors’ suggestions, finding no disagreements.

The authors defined the following concepts for results analysis: syntactic adaptation as the set of rules allowing different word options and associations to form sentences – i.e., how the terms in a sentence relate to one another; semantic adaptation as the equivalence of same-meaning words in American English and Brazilian Portuguese; and grammatical adaptation as the set of rules for the correct use of written and spoken language.

Three procedures were performed for the content validity evidence of the images selected to represent the original scale’s levels, following the recommendations of the Standards for Educational and Psychological Testing (2014)^(10)^. The initial procedure was a literature review to establish the operational definition of the term “pharyngeal residue.”

The second procedure was a visual-perceptual evaluation of 50 examinations of individuals with neurological etiologies from an image database at a reference center for the diagnosis of oropharyngeal dysphagia. These exams were randomly analyzed by the author of this study. From this analysis, 24 images were selected to represent six PR locations, divided into Region 1 (lateral pharyngeal wall, posterior pharyngeal wall, base of tongue, valleculae, and tip of epiglottis) and Region 2 (left lateral channel and left piriform recess, right lateral channel and right piriform recess, and postcricoid region). The images were also categorized into four levels of severity (no residue/residual coating; mild; moderate; and severe).

The third procedure was the visual-perceptual evaluation by three additional judges experienced with the examination. They answered a structured questionnaire indicating whether they “agreed,” “disagreed,” or had any suggestions for each selected image and location. The agreement among the judges was analyzed using Fleiss' Kappa test with a 95% confidence interval (95% CI).

## RESULTS

The results were presented in three stages. The first stage (S1) focused on the translation process and the instructions for using the scale, the second stage (S2) addressed the back-translation process, and the third stage (S3) covered the content validity of the images.

In S1, the 18 BRACS segments were divided for analysis. Nine had been translated identically, and nine diverged between T1 and T2. The consensus version maintained one of the nine discrepant segments as in T1, five as in T2, and the remaining ones were adjusted by consensus between the translators. The adjustments were made by comparing with the original version, aiming for the best equivalent term commonly used in Brazilian Portuguese. The results are detailed in Table 1.

**Table 1:** Translations 1 (T1) and 2 (T2), discrepancies between them, and adjustments made to the consensus BRACS version

BRACS	T1	T2	T1/T2
*1.Location and amount of residue*	Localização e quantidade de **resíduos**	Localização e quantidade de **resíduo**	Localização e quantidade de resíduos
*2.Mark all that apply*	**Indique** todas as alternativas que se aplicam	**Marque** todas as localizações que se aplicam	Marque todos os locais que se aplicam
*3.and then indicate the worst score attained from any location in the last row*	**Depois,** indique a pior pontuação **alcançada a partir de qualquer localização na última coluna**	**E, em seguida**, indique a pior pontuação **obtida em cada uma delas**	E, em seguida, indique a pior pontuação obtida em cada um deles
*4.Zone 1*	**Zona 1**	**Região 1**	Região 1
*5.Lateral pharyngeal Wall*	Parede lateral da faringe	Parede lateral da faringe	Parede lateral da faringe
*6.Posterior pharyngeal Wall*	Parede posterior da faringe	Parede posterior da faringe	Parede posterior da faringe
*7.Base of tongue*	Base da língua	Base da língua	Base da língua
*8.Valleculae*	Valéculas	Valéculas	Valéculas
*9.Tip of epiglottis*	Ponta da epiglote	Ponta da epiglote	Ponta da epiglote
*10.Zone 2*	**Zona 2**	**Região 2**	Região 2
*11.Left lateral channel and left piriform recess*	Canal lateral esquerdo e recesso piriforme esquerdo	Canal lateral esquerdo e recesso piriforme esquerdo	Canal lateral esquerdo e recesso piriforme esquerdo
*12.Right lateral channel and right piriform recess*	Canal lateral direito e recesso piriforme direito	Canal lateral direito e recesso piriforme direito	Canal lateral direito e recesso piriforme direito
*13.Postcricoid region*	Região pós-cricóidea	Região pós-cricóidea	Região pós-cricóidea
*14.Bolus 1*	**Bolo alimentar 1**	**Bolo 1**	Bolus alimentar 1
*15.None/coating*	Nenhum/**revestimento**	Nenhum	Nenhum resíduo/vestígio residual
*16.Mild*	**Ameno**	**Leve**	Leve
*17.Moderate*	Moderado	Moderado	Moderado
*18.Severe*	**Severo**	**Grave**	Grave

The words in bold in columns T1 and T2 indicate the discrepancies between translations 1 (T1) and 2 (T2)

**Caption: BRACS** = The Boston Residue and Clearance Scale; **T1** = translation 1; **T2** = translation 2; **T1/T2** = consensus version

Also, in S1, only one of the 14 segments in the instruction paragraph was translated identically between T1 and T2, while the other 13 diverged. These discrepancies were categorized as a) semantic, b) syntactic, and c) grammatical and were resolved by consensus, as shown in column 3 of Table 1.

In S2, Table 2 presents the BRACS translation, detailing the translation of 18 segments from American English into Brazilian Portuguese.

**Table 2:** Stage 1 translations 1 (T1) and (T2), discrepancies between them, and adjustments made to the consensus version of the BRACS’ instructions paragraph

PARAGRAPH	T1	T2	T1/T2
*1.BRACS is an 11-point ordinal scale measuring the severity of a residue problem*	A BRACS é uma escala ordinal de 11 pontos **cujo objetivo é mensurar** a gravidade de um problema de resíduos*	A BRACS é uma escala ordinal de 11 pontos **que avalia** a gravidade de resíduos faríngeos*	A BRACS é uma escala ordinal de 11 pontos que mensura o grau de comprometimento de resíduos faríngeos
*2.The scale specifically defines amount of residue*	Essa escala **indica, em específico,** a quantidade de resíduos **verificada**	A escala **determina, especificamente,** a quantidade de resíduos	A escala determina, em específico, a quantidade de resíduos faríngeos
*3.None/coating*	Nenhum resíduo/vestígio residual	Nenhum resíduo/vestígio residual	Nenhum resíduo/vestígio residual
*4.Mild = covering/filling <1/3 of the location*	Leve = **cobertura/preenchimento** **<1/3 do local**	Leve = **resíduos cobrindo/preenchendo** **<1/3 da localização**	Leve = resíduos cobrindo/preenchendo <1/3 do local
*5.Moderate Covering/filling 1/3-2/3 of the location*	Moderado = **cobertura/preenchimento 1/3-2/3 do local**	Moderado = **resíduos cobrindo/preenchendo 1/3-2/3 da localização**	Moderado = Resíduos cobrindo/preenchendo 1/3 – 2/3 do local
*6.Severe = covering/filling >2/3 of the location*	Grave = **cobertura/preenchimento** **>2/3 do local**	Grave = **resíduos cobrindo/preenchendo** **>2/3 da localização**	Grave = resíduos cobrindo/preenchendo >2/3 do local
*7.The amount of residue is scored in 12 locations in the laryngopharynx*	A quantidade de resíduos é pontuada **considerando** 12 locais na laringofaringe	A quantidade de resíduos é pontuada em 12 **localizações** na laringofaringe	A quantidade de resíduos é pontuada considerando 12 locais na laringofaringe
*8.An extra point is added if residue was noted in 4 or more anatomical regions*	**Um ponto extra é adicionado se o resíduo for observado em 4 ou mais regiões anatômicas**	**Se houver resíduos em 4 ou mais regiões anatômicas, um ponto extra é adicionado**	Se houver resíduos em quatro ou mais regiões anatômicas, adicione um ponto à somatória
*9.An additional point is added if residue was ever present inside the vestibule, placing the individual at highest risk for aspiration after the swallow*	**Um segundo ponto extra é adicionado se o resíduo for observado dentro do vestíbulo, o que indica que o indivíduo apresenta maior risco de aspiração após a deglutição**	**Se houver resíduos no interior do vestíbulo, colocando o indivíduo em maior risco de aspiração após a deglutição, um ponto extra é adicionado**	Se houver resíduos no interior do vestíbulo, colocando o indivíduo em maior risco de aspiração após a deglutição, adicione um ponto à somatória
*10.If residue is observed and the individual demonstrates no spontaneous clearing swallows, and extra point is added to account for the apparent lack of pharyngeal sensation*	Se **for observado resíduo**, e o indivíduo não **demonstrar deglutição espontânea, um terceiro ponto extra é adicionado para indicar uma aparente ausência de** sensação faríngea	Se **forem observados resíduos** e o indivíduo não **manifestar deglutições espontâneas, um** **ponto extra é adicionado para explicar a aparente falta** de sensação faríngea	Se houver resíduos e o indivíduo não manifestar deglutições espontâneas, adicione um ponto à somatória, considerando que, aparentemente, existe a falta de sensação faríngea
*11.Cued or spontaneous swallows are then judged for effectiveness*	**Então, a deglutição espontânea ou a deglutição estimulada são classificadas quanto à eficácia**	**As deglutições espontâneas ou estimulada são, então, julgadas quanto à sua eficácia**	Por fim, as deglutições espontâneas ou estimuladas são, então, julgadas quanto a sua eficiência
*12.Yes = 80-100% cleared*	Sim (**eficaz**) = 80-100% de **resíduos eliminados**	Sim = **limpeza de** 80- 100% dos resíduos	Sim = limpeza de 80-100% dos resíduos
*13.Partially = 20-80% cleared*	Parcialmente **eficaz** = 20- 80% de **resíduos eliminados**	Parcialmente = **limpeza de** 20-80% dos resíduos	Parcialmente = limpeza de 20-80% dos resíduos
*14.No = 0-20% cleared*	Não (**não eficaz**) = 0-20% de **resíduos eliminados**	Não = **limpeza de** 0-20% dos resíduos	Não = limpeza de 0-20% dos resíduos

* The words in bold in columns T1 and T2 indicate the discrepancies between translations 1 (T1) and 2 (T2)

**Caption:T1** = translation 1; **T2** = translation 2; **T1/T2** = consensus version

In the BRACS back-translation stage, 18 segments were back-translated from Brazilian Portuguese to American English, as shown in Table 3. Of the total segments, 11 were translated in agreement between B1 and B2, while seven diverged. One of the latter was adjusted to align with B1, and six were adjusted to align with B2. Discrepancies were resolved in the consensus version, as shown in the same table.

**Table 3:** Back translations 1 (B1) and 2 (B2), discrepancies between them, and adjustments made to the consensus BRACS version

BRACS	B1	B2	B1/B2
1.Localização e quantidade de resíduos	*Location and quantity of residue*	*Location and* ***amount*** *of residue*	*Location and amount of residue*
2.Marque todos os locais que se aplicam	*Check all* ***sites*** *that apply*	*Check all* ***locations*** *that apply*	*Check all locations that apply*
3.e, em seguida, indique a pior pontuação obtida em cada um deles	*and then indicate the worst score obtained for each* ***of them***	*and then indicate the worst score obtained in each*	*and then indicate the worst score obtained in each*
4.Região 1	*Region 1*	*Region 1*	*Region 1*
5.Parede lateral da faringe	*Lateral wall of the pharynx*	*Lateral wall of pharynx*	*Lateral wall of the pharynx*
6.Parede posterior da faringe	*Posterior wall of the pharynx*	*Posterior wall of the pharynx*	*Posterior wall of the pharynx*
7.Base da língua	*Base of the tongue*	*Base of the tongue*	*Base of the tongue*
8.Valéculas	***Vallecula***	***Valleculae***	*Valleculae*
9.Ponta da epiglote	*Tip of the epiglottis*	*Tip of the epiglottis*	*Tip of the epiglottis*
10.Região 2	*Region 2*	*Region 2*	*Region 2*
11.Canal lateral esquerdo e recesso piriforme esquerdo	*Left lateral canal and left* ***pyriform*** *recess*	*Left lateral canal and left* ***piriform*** *recess*	*Left lateral canal and left* ***pyriform*** *recess*
12.Canal lateral direito e recesso piriforme direito	*Right lateral canal and right* ***pyriform*** *recess*	*Right lateral canal and right* ***piriform*** *recess*	*Right lateral canal and right pyriform recess*
13.Região pós- cricóide	*Postcricoid region*	*Postcricoid region*	*Postcricoid region*
14.Bolus alimentar 1	*Food bolus*	*Food Bolus*	*Food Bolus*
15.Nenhum resíduo/vestígio residual	*No residue/residual waste*	*No residue/traces*	*No residue/traces*
16.Leve	***Light***	***Mild***	*Mild*
17.Moderado	*Moderate*	*Moderate*	*Moderate*
18.Grave	*Severe*	*Severe*	*Severe*

The words in bold in columns B1 and B2 indicate the discrepancies between back-translations 1 (B1) and 2 (B2)

**Caption:B1** = back translation 1; **B2** = back translation 2; **B1/B2** = consensus version

The back-translation of the instruction paragraph had the same number of agreements and discrepancies as in the translation. B1 and B2 agreed in only one segment and diverged in the remaining 13, as shown in Table 4. Discrepancies were resolved in the consensus version, shown in the same table.

**Table 4:** Back translations 1 (B1) and 2 (B2), discrepancies between them, and adjustments made to the consensus version of the BRACS’ instructions paragraph

PARAGRAPH	B1	B2	B1/B2
1.A BRACS é uma escala ordinal de 11 pontos que mensura o grau de comprometimento de resíduos faríngeos	*BRACS (The Boston Residue and Clearance Scale) is an 11-point ordinal scale that measures the degree of* ***compromisse*** *of pharyngeal residue*	***The*** *BRACS (Boston Residue and Clearance Scale) is an 11-point ordinal scale that measures the degree of* ***impairment*** *of pharyngeal residue*	*The BRACS (Boston Residue and Clearance Scale) is na 11-point ordinal scale that measures the degree of impairment of pharyngeal residue*
2.A escala determina, em específico, a quantidade de resíduos faríngeos	*The scale specifically determines the amount of pharyngeal residue*	*The scale specifically determines the amount of pharyngeal residue*	*The scale specifically determines the amount of pharyngeal residue*
3.Nenhum resíduo/vestígio residual	*No residue/* ***vestigial residue***	*No residue/* ***traces***	*No residue/traces*
4.Leve = resíduos cobrindo/preenchendo <1/3 do local	***light*** *= residue covering/filling <1/3 of the* ***site***	***mild*** *= residue covering/filling <1/3 of the* ***location***	*mild = residue covering/filling <1/3 of the location*
5.Moderado = Resíduos cobrindo/preenchendo 1/3 – 2/3 do local	*moderate = residue covering/filling 1/3 – 2/3 of the* ***site***	*moderate = residue covering/filling 1/3 – 2/3 of the* ***location***	*moderate = residue covering/filling 1/3 – 2/3 of the location*
6.Grave = resíduos cobrindo/preenchendo >2/3 do local	*severe = residue covering/filling >2/3 of the* ***site***	*severe = residue covering/filling >2/3 of the* ***location***	*severe = residue covering/filling >2/3 of the location*
7.A quantidade de resíduos é pontuada considerando 12 locais da laringofaringe	*The amount of residues* ***punctuated*** *in 12* ***locations of laryngopharynx***	*The amount of residue is* ***scored*** *at 12* ***laryngopharyngeal sites***	*The amount of residue is scored at 12 laryngopharyngeal locations*
8.Se houver resíduos em quatro ou mais regiões anatômicas, adicione um ponto à somatória	***If there is residue in four or more anatomical regions, na extra point must be added to the sum***	***An extra point is added to the sum if residue is present in four or more anatomical regions***	*If there is residue in four or more anatomical regions, na extra point must be added to the sum*
9.Se houver resíduos no interior do vestíbulo, colocando o indivíduo em maior risco de aspiração após a deglutição, adicione um ponto à somatória	***If there is residue inside the vestibule, placing the individual at increased risk of aspiration after swallowing, another extra point must be added to the sum***	***Another point is added to the sum if there is residue inside the vestibule, putting the individual at greater risk of aspiration after swallowing***	*If there is residue inside the vestibule, placing the individual at increased risk of aspiration after swallowing, another extra point must be added to the sum*
10.Se houver resíduos e o indivíduo não manifestar deglutições espontâneas, adicione um ponto à somatória, considerando que, aparentemente, existe a falta de sensação faríngea	***And if there is residue and the individual does not manifest spontaneous swallows, another extra point must be added to the sum, considering that, apparently, there is a*** *lack of pharyngeal sensation*	***An additional point is added to the sum if there is residue and the individual does not manifest spontaneous swallowing, indicating an apparent*** *lack of pharyngeal sensation*	*And if there is residue and the individual does not manifest spontaneous swallows,another extra point must be added to the sum, indicating na apparent lack of pharyngeal sensation*
11.Por fim, as deglutições espontâneas ou estimuladas são, então, julgadas quanto a sua eficiência	*Finally, spontaneous or stimulated swallows are judged* ***by*** *their efficiency*	*Finally, spontaneous or stimulated swallows are judge* ***according to*** *their efficiency*	*Finally, spontaneous or stimulated swallows are judge according to their efficiency*
12.Sim = limpeza de 80-100% dos resíduos	*Yes =* ***cleaning*** *80-100% of residue*	*Yes = 80-100% of residue* ***cleared***	*Yes = 80-100% of residue cleared*
13.Parcialmente = limpeza de 20-80% dos resíduos	*Partially =* ***cleaning*** *of 20-80% of residue*	*Partially = 20-80% of residue* ***cleared***	*Partially = 20-80% of residue cleared*
14.Não = limpeza de 0-20% dos resíduos	*No =* ***clearing of*** *0-20% of residue*	*No = 0-20% of residue* ***cleared***	*No = 0-20% of residue cleared*

The words in bold in columns B1 and B2 indicate the discrepancies between back-translations 1 (B1) and 2 (B2)

**Caption:B1** = back translation 1; **B2** = back translation 2; **B1/B2** = consensus version

After completing this stage, following the translators' suggestions and the study authors' decision, the back-translated BRACS was compared with the original version to confirm the equivalencies. This process was carried out in an online meeting, discussing semantic and grammatical issues related to the anatomy of the regions in question; no discrepancies were found.

Lastly, S3 addressed the content validity results for the selection of images. The agreement among the judges was 0.958, with a confidence interval of 0.051 and 0.036, suggesting almost perfect agreement between them. The judges had three discrepancies in Regions 1 and 2 concerning the locations and severity levels, as follows: Lateral pharyngeal wall and posterior pharyngeal wall – Mild; Valleculae and the tip of epiglottis – Moderate; Postcricoid region – Moderate.

Regarding the first location discrepancy, one judge suggested that the chosen image represented the lateral pharyngeal wall, rather than both the lateral and posterior pharyngeal walls. In the second location discrepancy, valleculae and the tip of epiglottis, another judge recommended including more images of the tip of epiglottis to better differentiate severity levels (mild and severe). Lastly, the judges commented on the difficulty in assessing the severity level from the static postcricoid image, leading to uncertainty between moderate and severe. The authors considered the discrepancies and maintained by consensus the selected images as representative of the severity levels.

The images agreed on to represent each region and level as previously proposed in the BR-BRACS are displayed in Appendix A.

## DISCUSSION

A simple literature review can easily detect the scarcity of tools to assess and classify the degree of impairment in oropharyngeal dysphagia with evidence of validity and developed in Brazilian Portuguese. The translation and adaptation of an instrument available in another language is often a solution to this problem, provided it follows an appropriate method^(11)^. Moreover, the translation process varies considerably in guidelines and recommendations, and many authors adapt the stages by combining one or more methods^(12-15)^.

Semantic and syntactic divergences in translation and back-translation are common. In the case of the BR-BRACS, they were more frequent in the translation of the instruction paragraph than in the scale itself. This issue is easily understood when compared with other translations of scales based on anatomical regions, as discrepancies are rarely found in human anatomy terms^(16-18)^. Translations of longer sentences are more likely to present divergences, as also noted in other translation studies, whose authors reported difficulties in analyzing semantic, idiomatic, conceptual, linguistic, and contextual discrepancies^(19-25)^.

In addition to sentence length and its impact on translations with syntactic divergences, there were semantic issues related to the correct healthcare terms. This technical aspect of translations, as highlighted in other studies, often arises due to cross-cultural adaptation – e.g., translating “cookie” as “*bolacha recheada*” (“sandwich cookie”)^(12)^ – and difficulties with items that lack an approximate translation in the target language with the same meaning intended by the original author^(20)^.

The discussion on translations, particularly the synthesis of translations involving the study authors, points out the fact that few analyzed articles reported the original scale authors’ participation in translation synthesis and joint analysis with the translators. On the other hand, they emphasized the importance of training and/or years of experience in translation or work on the topic at hand^(24-26)^.

The translators in this study had an average of over 5 years of experience in translation. Following the steps described by Beaton et al.^(9)^, one of the translators responsible for the first stage was familiar with the technical concepts of the instrument, demonstrating expertise in the field. The results of the translation and back-translation stages were as expected, yielding a translation that reflected the language and its semantic, syntactic, and grammatical aspects. Lastly, it is understood that translation and back-translation alone do not provide sufficient evidence of validity. In other words, although the final translation model resulted in a translated and back-translated scale suitable for clinical application, much further evidence is necessary to ensure the instrument’s validity and reliability for its intended purpose^(15)^.

Furthermore, the original version of the scale did not include representative images to facilitate training for the professionals administering it. Hence, it was necessary to verify content validity evidence for the selection and inclusion of images and thus proceed with the investigation of validity evidence for this scale. This step was crucial to enable its reliable future use by both researchers and clinicians.

The discrepancies cited in the results highlighted various factors that complicated the selection of images for the translated instrument. There was greater difficulty in regions encompassing more than one location, such as “Lateral and posterior pharyngeal wall” and “Valleculae and tip of epiglottis,” as many of the analyzed exams did not show both structures with residue. The judges also cited the evaluation of static images, such as that of the “Postcricoid region.” According to one of the judges, the image would be challenging to classify due to potential interference from the posterior pharyngeal wall, leading to uncertainty when distinguishing between the “moderate” and “severe” grades.

The BRACS assesses PR in different regions of the pharynx, including the efficiency of the clearance mechanism (i.e., residue removal), which sets it apart from other scales^(6,8)^. Moreover, it offers clear PR level markers, making it a strong instrument to support clinical decision-making in oropharyngeal dysphagia, alongside other clinical and instrumental findings.

On the other hand, the absence of images in the original BRACS hinders the precise understanding of the original authors' expectations regarding the visual elements necessary for training and applying the scale. This allowed us to advance and propose images that could facilitate this process.

Other PR scales^(6,8)^ have often used visual representation with selected static images to depict swallowing findings and classify impairment levels. Although the images are static, PR regards the volume and anatomical markers of the regions involved, indicating differences between levels. However, it is important to emphasize that every qualitative and quantitative swallowing analysis method always requires training for the evaluator to minimize analysis subjectivity and ensure instrument reliability.

Hence, this stage of the process has been completed, and it is necessary to proceed to confirm the instrument’s advantages and disadvantages in measuring PR in FEES. Therefore, new steps will be taken towards future contributions to this diagnostic method for classifying PR in oropharyngeal dysphagia.

## CONCLUSION

The process of translating and cross-culturally adapting the scale has been completed, and the selection of images for the visual-perceptual evaluation of PR in the BR-BRACS has been concluded with evidence of content validity.

## Figures and Tables

**Figure:**
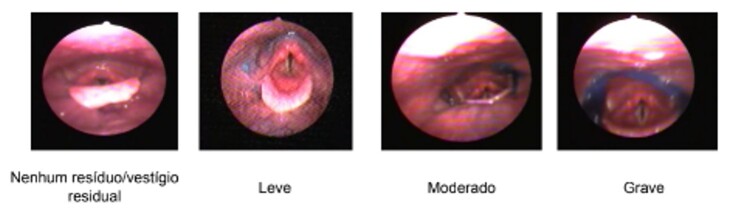
Parede lateral da faringe e parede posterior da faringe

**Figure:**
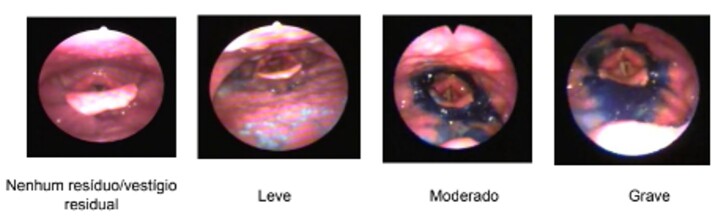
Base de língua

**Figure:**
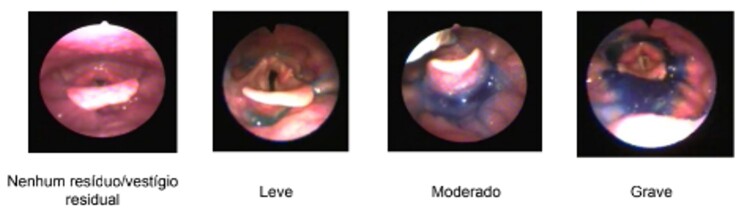
Valéculas, ponta da epiglote

**Figure:**
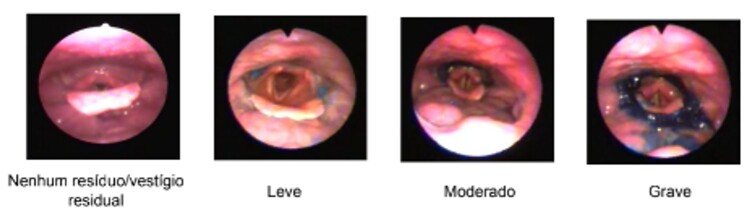
Canal lateral e recesso piriforme esquerdo e/ou direito

**Figure:**
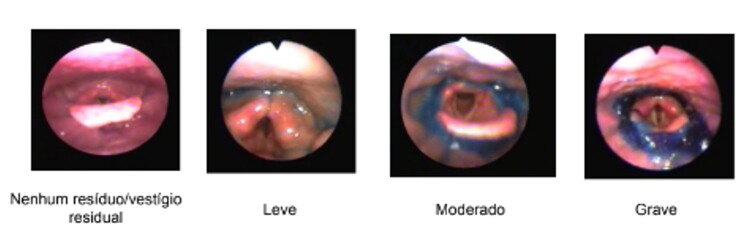
Região pós-cricóidea

## Apêndice A Modelo da estruturação da escala BR-BRACS com as imagens selecionadas

A BRACS (The Boston Residue and Clearance Scale) é uma escala ordinal de 11 pontos que mensura o grau de comprometimento dos resíduos faríngeos. A escala determina, especificamente, a quantidade de resíduos faríngeos (nenhum resíduo/vestígio residual; leve = resíduos cobrindo/preenchendo <1/3 do local; moderado = resíduos cobrindo/preenchendo 1/3 - 2/3 do local; grave = resíduos cobrindo/preenchendo >2/3 do local). A quantidade de resíduos é pontuada em 12 locais da laringofaringe.

Se houver resíduos em quatro ou mais regiões anatômicas, um ponto extra deve ser adicionado à somatória. Se houver resíduos no interior do vestíbulo, colocando o indivíduo em maior risco de aspiração após a deglutição, outro ponto extra deve ser adicionado à somatória. E, se houver resíduos e o indivíduo não manifestar deglutições espontâneas, outro ponto extra deve ser adicionado à somatória, considerando que, aparentemente, há falta de sensação faríngea.

Por fim, as deglutições espontâneas ou estimuladas são julgadas quanto a sua eficiência (sim = limpeza de 80 - 100% dos resíduos; parcialmente = limpeza de 20 - 80% dos resíduos; não = limpeza de 0 - 20% dos resíduos).

**Table:**

Localização e quantidade de resíduos – Marque todos os locais que se aplicam e, em seguida, indique a pior pontuação obtida em cada um deles.	Bolus alimentar
	Nenhum resíduo/vestígio residual Leve Moderado Grave
**Região 1**	
Parede lateral da faringe, parede posterior da faringe	
Base da língua	
Valéculas, ponta da epiglote	
**Região 2**	
Canal lateral esquerdo e recesso piriforme esquerdo	
Canal lateral direito e recesso piriforme direito	
Região pós-cricóidea	
