# Supplementary material for: Translation and cross-cultural adaptation of the Brazilian version of the Boston Residue and Clearance Scale (BR-BRACS)
Source: Codas. 2025 Aug 8;37(4):e20240256. doi: 10.1590/2317-1782/e20240256en (PMC12337712; doi:10.1590/2317-1782/e20240256en)
Supplement: Translation

# Translation: Tradução e adaptação transcultural da versão brasileira da Boston Residue and Clearance Scale (BR-BRACS)

**DOI:** 10.1590/2317-1782/e20240256pt

**Authors:** Giulia Beatriz Pozena Scaranelo, Suely Mayumi Motonaga Onofri, Leandro de Araújo Pernambuco, Roberta Gonçalves da Silva

**Affiliations:** 1 Laboratório de Disfagia e Programa de Pós-Graduação em Ciências da Saúde e Comunicação Humana do Departamento de Fonoaudiologia , Universidade Estadual Paulista "Júlio de Mesquita Filho" - UNESP - Marília (SP), Brasil.; 2 Departamento de Fonoaudiologia e Programa de Pós-Graduação em Saúde da Comunicação Humana da Universidade Federal de Pernambuco-UFPE, Recife/PE, Brasil.

**Keywords:** Tradução, Comparação Transcultural, Transtornos de Deglutição, Avaliação, Endoscopia

## Abstract

**Objetivo:**

Este estudo teve por objetivo traduzir e adaptar transculturalmente para a Língua Portuguesa do Brasil a The Boston Residue and Clearance Scale (BR-BRACS) e validar o conteúdo da seleção de imagens para compor a escala.

**Método:**

Projeto aprovado pelo Comitê de Ética da Instituição sob o número 67715717.6.0000.5406. Foi realizada a tradução para o idioma alvo, síntese das traduções e retrotradução para o idioma original. Para a validade de conteúdo das imagens representativas da escala foi realizada busca na literatura para definir o conceito de resíduos faríngeos, análise de 50 exames de videoendoscopia da deglutição e análise perceptivo visual das imagens por três juízes. A concordância entre juízes foi analisada por meio do teste Kappa Fleiss com intervalo de confiança de 95%.

**Resultados:**

Na tradução houve discrepâncias no contexto lexical e sintático que foram ajustadas em formato de consenso entre tradutores e autores, e na retrotradução as versões apresentaram equivalência. As imagens propostas para compor a escala apresentaram concordância quase perfeita entre os juízes.

**Conclusão:**

O processo de tradução e adaptação transcultural, bem como a seleção das imagens para análise perceptivo-visual de resíduos faríngeos da BR-BRACS foi concluído com evidência de validade de conteúdo.

## INTRODUÇÃO

O processo de avaliação na área da saúde, clínica ou por meios de distintos exames, necessita de procedimentos, protocolos e escalas confiáveis e com acurácia diagnóstica. Em relação aos diagnósticos de transtornos de deglutição, os instrumentos de avaliação com escalas que mensuram o grau de comprometimento são importantes tanto para a compreensão dos níveis de prejuízo na biomecânica da deglutição, quanto para analisar a natureza e o prognóstico do problema em qualquer momento do atendimento do paciente. Tais instrumentos, sejam eles de avaliação clínica ou exames instrumentais, são complementares e excelentes meios de *feedback* aos familiares e pacientes, além de serem parte do conjunto de marcadores que auxiliam o profissional na tomada de decisão clínica^(1,2)^.

Na área de disfagia orofaríngea, a presença de resíduos faríngeos (RF) é considerada marcador de risco para aspiração laringotraqueal e apesar da existência de diversas escalas para análise de RF, essas foram publicadas na Língua Inglesa Americana e, na grande maioria, destinadas à classificação de RF no exame de Videofluoroscopia da Deglutição (VFD)^(3-5)^ e apenas algumas para análise em Videoendoscopia de Deglutição^(6-8)^. Visando potencializar a análise de RF por meio da Videoendoscopia da Deglutição (VED), considerado um excelente exame instrumental para a avaliação desse achado, a *The Boston Residue and Clearance Scale* (BRACS) é uma das escalas disponíveis para mensurar os níveis de comprometimento deste achado faríngeo.

Assim, a tradução e adaptação de escalas para classificação de RF para a Língua Portuguesa do Brasil demonstra-se necessária para potencializar o diagnóstico e a tomada de decisão clínica apoiada em marcadores reconhecidos internacionalmente. Portanto, este estudo teve por objetivo traduzir e adaptar transculturalmente para a Língua Portuguesa do Brasil a *The Boston Residue and Clearance Scale* (BR-BRACS) e validar o conteúdo da seleção de imagens para compor a escala.

## MÉTODO

Este estudo foi aprovado pelo Comitê de Ética em Pesquisa (CEP) sob o número de protocolo 67715717.6.0000.5406. Para iniciar este estudo a autora principal do instrumento foi contactada por e-mail e autorizou a realização. A BRACS é uma escala ordinal de 11 pontos, dividida em 12 locais com quatro diferentes classificações de quantidade de resíduo (Nenhum/revestimento, Leve, Moderado e Grave). Os locais de identificação de resíduos foram divididos em duas regiões, sendo a Região 1 composta pela Parede lateral da faringe, parede posterior da faringe, base da língua, valéculas, ponta de epiglote e a Região 2 composta pelo Canal lateral esquerdo e recesso piriforme esquerdo, canal lateral direito e recesso piriforme direito e região pós cricóidea. A pontuação da escala é realizada de acordo com a presença de resíduos em um ou mais locais e ao final classifica-se a eficiência da deglutição com base em deglutições estimuladas ou espontâneas para limpeza dos resíduos^(7)^.

Para o processo de tradução e adaptação transcultural da *The Boston Residue and Clearance Scale,* aplicou-se a metodologia recomendada por Beaton et al.^(9)^, que contempla seis etapas e nesta fase do estudo foram realizadas as três primeiras, sendo elas: (1) Tradução para o idioma alvo, a Língua Portuguesa do Brasil; (2) Síntese das Traduções e (3) Retrotradução para o idioma original, a Língua Inglesa Norte-Americana, descritas a seguir:

### Etapa 1 – Tradução para o idioma alvo, no caso a Língua Portuguesa do Brasil

Realizou-se o processo de tradução do instrumento da língua original (Inglês Norte-Americano) para a Língua Portuguesa do Brasil, por duas tradutoras experientes em traduções para estudos científicos na área da saúde e inclusive de outros instrumentos para disfagia orofaríngea. Os critérios para seleção dos tradutores incluíram ser nativo Brasileiro, fluente no idioma original do instrumento e ter experiência com tradução na área de saúde. As tradutoras apresentaram média de 9 anos de experiência na área e preencheram todos os critérios. Após serem contatadas, foi enviado por e-mail, o arquivo contendo o parágrafo de instruções e a escala *“The Boston Residue and Clearance Scale* (BRACS)*”* a ser traduzido no prazo de uma semana. Ambas realizaram a tradução individualmente, gerando duas traduções (T1 e T2).

Ainda neste processo, realizou-se a tradução do parágrafo de instruções para o uso e pontuação da escala BRACS, que foi selecionado do artigo original e dividido em 14 segmentos enviados às tradutoras para o registro de suas traduções. Após as traduções, o parágrafo e a escala seguiram para a etapa 2.

### Etapa 2 – Síntese das traduções

O comitê de pesquisadores, formado pelos autores, com média de 15 anos de experiência na área do instrumento a ser traduzido, e os dois tradutores da etapa 1 se reuniram virtualmente com o objetivo de eliminar discrepâncias no contexto lexical e sintático e elaborar a versão consenso (T1+T2) na Língua Portuguesa do Brasil. A reunião foi realizada por meio da plataforma *Google Meet*, onde pela primeira vez as tradutoras visualizaram juntas suas traduções. Os ajustes foram realizados por consenso entre as tradutoras e autores deste estudo, e a comparação com a versão original, buscando o melhor termo equivalente e usualmente utilizado na área de especialidade na Língua Portuguesa do Brasil.

### Etapa 3 – Retrotradução para o idioma original e adaptação transcultural - Língua Inglesa Norte-Americana

Realizou-se o processo de Retrotradução (*back-translation*) da versão consenso (T1+2) em Língua Portuguesa do Brasil para o idioma original do instrumento (Inglês Norte-Americano) por dois tradutores nativo americanos e fluentes na Língua Portuguesa do Brasil. Após o processo, as discrepâncias da versão retrotraduzida foram discutidas em reunião de consenso entre tradutores e os pesquisadores, gerando a versão final.

Ao final da etapa optou-se por comparar a versão consenso com a versão original conforme sugestões dos tradutores e autores do estudo, onde não houveram discordâncias.

Para a análise dos resultados, os autores definiram os conceitos de adaptação sintática como o conjunto de regras que permitem diferentes possibilidades e associação de palavras para a formulação da frase, ou seja, como os termos dentro da frase relacionam-se entre si; adaptação semântica equivalência entre as palavras com o mesmo significado nas Línguas Inglesa Norte-Americana e Português do Brasil e adaptação gramatical como o conjunto de regras para o uso correto da língua escrita e falada.

Para a evidência de validade de conteúdo das imagens selecionadas para compor os níveis propostos pela escala original foram realizados três procedimentos, conforme recomendação do *Standards for Educational and Psychological Testing (2014)* ^(10)^. O procedimento inicial constou de busca na literatura científica para a definição operacional do termo resíduos faríngeos.

O segundo procedimento constou da análise perceptivo visual de 50 exames do banco de imagens de população com etiologia neurológica, de um centro de referência no diagnóstico de disfagia orofaríngea, analisados aleatoriamente pelo autor deste estudo. Foram selecionadas 24 imagens para representar seis localizações de resíduos faríngeos divididas em Região 1 (Parede lateral da faringe, Parede posterior da faringe; Base da língua e Valéculas, ponta da epiglote) e Região 2 (Canal lateral esquerdo e recesso piriforme esquerdo; Canal lateral direito e recesso piriforme direito e Região pós-cricóidea), além de quatro classificações de grau de comprometimento (nenhum resíduo/vestígio residual; leve; moderado e grave).

O terceiro procedimento constou da análise perceptivo visual de três outros juízes experientes no exame, que responderam no questionário estruturado se “concordavam”, “discordavam” ou apresentavam alguma sugestão para cada imagem e localização selecionada. A concordância entre juízes foi analisada por meio do teste Kappa Fleiss com intervalo de confiança de 95% (IC 95%).

## RESULTADOS

Os resultados foram apresentados em três etapas. A primeira etapa (E1) referente ao processo de tradução e instruções do uso da escala, a segunda sobre o processo de retrotradução (E2) e a terceira sobre a validade de conteúdo das imagens (E3).

Na E1, foram divididos 18 segmentos da escala BRACS, onde nove foram traduzidos do mesmo modo entre as traduções 1 e 2 (T1 e T2) e nove mostraram-se divergentes entre as T1 e T2. Na versão consenso, dos nove segmentos divergentes entre as T1 e T2, optou-se por manter um como na T1, cinco como na T2, e o restante foi reajustado por consenso entre as tradutoras. As adequações foram feitas por consenso entre as tradutoras e a comparação com a versão original, buscando o melhor termo equivalente e usualmente utilizado na Língua Portuguesa do Brasil. Os resultados estão descritos na Tabela 1.

**Tabela 1:** Tradução entre T1 e T2, divergências e ajustes na versão consenso da escala BRACS

BRACS	T1	T2	T1/T2
*1.Location and amount of residue*	Localização e quantidade de **resíduos**	Localização e quantidade de **resíduo**	Localização e quantidade de resíduos
*2.Mark all that apply*	**Indique** todas as alternativas que se aplicam	**Marque** todas as localizações que se aplicam	Marque todos os locais que se aplicam
*3.and then indicate the worst score attained from any location in the last row*	**Depois,** indique a pior pontuação **alcançada a partir de qualquer localização na última coluna**	**E, em seguida**, indique a pior pontuação **obtida em cada uma delas**	E, em seguida, indique a pior pontuação obtida em cada um deles
*4.Zone 1*	**Zona 1**	**Região 1**	Região 1
*5.Lateral pharyngeal Wall*	Parede lateral da faringe	Parede lateral da faringe	Parede lateral da faringe
*6.Posterior pharyngeal Wall*	Parede posterior da faringe	Parede posterior da faringe	Parede posterior da faringe
*7.Base of tongue*	Base da língua	Base da língua	Base da língua
*8.Valleculae*	Valéculas	Valéculas	Valéculas
*9.Tip of epiglottis*	Ponta da epiglote	Ponta da epiglote	Ponta da epiglote
*10.Zone 2*	**Zona 2**	**Região 2**	Região 2
*11.Left lateral channel and left piriform recess*	Canal lateral esquerdo e recesso piriforme esquerdo	Canal lateral esquerdo e recesso piriforme esquerdo	Canal lateral esquerdo e recesso piriforme esquerdo
*12.Right lateral channel and right piriform recess*	Canal lateral direito e recesso piriforme direito	Canal lateral direito e recesso piriforme direito	Canal lateral direito e recesso piriforme direito
*13.Postcricoid region*	Região pós-cricóidea	Região pós-cricóidea	Região pós-cricóidea
*14.Bolus 1*	**Bolo alimentar 1**	**Bolo 1**	Bolus alimentar 1
*15.None/coating*	Nenhum/**revestimento**	Nenhum	Nenhum resíduo/vestígio residual
*16.Mild*	**Ameno**	**Leve**	Leve
*17.Moderate*	Moderado	Moderado	Moderado
*18.Severe*	**Severo**	**Grave**	Grave

As palavras grifadas em negrito nas colunas T1 e T2 referem-se às divergências entre as traduções 1 (T1) e 2 (T2)

**Legenda:BRACS** = *The Boston Residue and Clearance Scale*; **T1** = Tradução 1; **T2** = Tradução 2; **T1/T2** = Versão consenso

Ainda na E1, dos 14 segmentos do parágrafo de instruções, apenas um foi traduzido do mesmo modo entre as T1 e T2 e os outros 13 apresentaram divergências. As divergências encontradas nesta etapa foram categorizadas em a) semânticas, b) sintáticas e c) gramaticais. As divergências foram resolvidas por consenso conforme mostra a coluna 3 da Tabela 1.

Já na E2, na Tabela 2 está apresentada a tradução da escala BRACS, com tradução de 18 segmentos da Língua Inglesa Norte-Americana para a Língua Portuguesa do Brasil.

**Tabela 2:** Traduções da etapa 1 entre T1 e T2, divergências e ajustes na versão consenso do parágrafo de instruções da escala BRACS

PARÁGRAFO	T1	T2	T1/T2
*1.BRACS is an 11-point ordinal scale measuring the severity of a residue problem*	A BRACS é uma escala ordinal de 11 pontos **cujo objetivo é mensurar** a gravidade de **um problema de resíduos**	A BRACS é uma escala ordinal de 11 pontos **que avalia** a gravidade de **resíduos faríngeos**	A BRACS é uma escala ordinal de 11 pontos que mensura o grau de comprometimento de resíduos faríngeos
*2.The scale specifically defines amount of residue*	Essa escala **indica, em específico,** a quantidade de resíduos **verificada**	A escala **determina, especificamente,** a quantidade de resíduos	A escala determina, em específico, a quantidade de resíduos faríngeos
*3.None/coating*	Nenhum resíduo/vestígio residual	Nenhum resíduo/vestígio residual	Nenhum resíduo/vestígio residual
*4.Mild = covering/filling <1/3 of the location*	Leve = **cobertura/preenchimento <1/3 do local**	Leve = **resíduos cobrindo/preenchendo <1/3 da localização**	Leve = resíduos cobrindo/preenchendo <1/3 do local
*5.Moderate Covering/filling 1/3-2/3 of the location*	Moderado = **cobertura/preenchimento 1/3-2/3 do local**	Moderado = **resíduos cobrindo/preenchendo 1/3-2/3 da localização**	Moderado = Resíduos cobrindo/preenchendo 1/3 – 2/3 do local
*6.Severe = covering/filling >2/3 of the location*	Grave = **cobertura/preenchimento >2/3 do local**	Grave = **resíduos cobrindo/preenchendo >2/3 da localização**	Grave = resíduos cobrindo/preenchendo >2/3 do local
*7.The amount of residue is scored in 12 locations in the laryngopharynx*	A quantidade de resíduos é pontuada **considerando** 12 locais na laringofaringe	A quantidade de resíduos é pontuada em 12 **localizações** na laringofaringe	A quantidade de resíduos é pontuada considerando 12 locais na laringofaringe
*8.An extra point is added if residue was noted in 4 or more anatomical regions*	**Um ponto extra é adicionado se o resíduo for observado em 4 ou mais regiões anatômicas**	**Se houver resíduos em 4 ou mais regiões anatômicas, um ponto extra é adicionado**	Se houver resíduos em quatro ou mais regiões anatômicas, adicione um ponto à somatória
*9.An additional point is added if residue was ever present inside the vestibule, placing the individual at highest risk for aspiration after the swallow*	**Um segundo ponto extra é adicionado se o resíduo for observado dentro do vestíbulo, o que indica que o indivíduo apresenta maior risco de aspiração após a deglutição**	**Se houver resíduos no interior do vestíbulo, colocando o indivíduo em maior risco de aspiração após a deglutição, um ponto extra é adicionado**	Se houver resíduos no interior do vestíbulo, colocando o indivíduo em maior risco de aspiração após a deglutição, adicione um ponto à somatória
*10.If residue is observed and the individual demonstrates no spontaneous clearing swallows, and extra point is added to account for the apparent lack of pharyngeal sensation*	Se **for observado resíduo**, e o indivíduo não **demonstrar deglutição espontânea, um terceiro ponto extra é adicionado para indicar uma aparente ausência de** sensação faríngea	Se **forem observados resíduos** e o indivíduo não **manifestar deglutições espontâneas, um ponto extra é adicionado para explicar a aparente falta** de sensação faríngea	Se houver resíduos e o indivíduo não manifestar deglutições espontâneas, adicione um ponto à somatória, considerando que, aparentemente, existe a falta de sensação faríngea
*11.Cued or spontaneous swallows are then judged for effectiveness*	**Então, a deglutição espontânea ou a deglutição estimulada são classificadas quanto à eficácia**	**As deglutições espontâneas ou estimulada são, então, julgadas quanto à sua eficácia**	Por fim, as deglutições espontâneas ou estimuladas são, então, julgadas quanto a sua eficiência
*12.Yes = 80-100% cleared*	Sim (**eficaz**) = 80-100% de **resíduos eliminados**	Sim = **limpeza de** 80- 100% dos resíduos	Sim = limpeza de 80-100% dos resíduos
*13.Partially = 20-80% cleared*	Parcialmente **eficaz** = 20- 80% de **resíduos eliminados**	Parcialmente = **limpeza de** 20-80% dos resíduos	Parcialmente = limpeza de 20-80% dos resíduos
*14.No = 0-20% cleared*	Não (**não eficaz**) = 0-20% de **resíduos eliminados**	Não = **limpeza de** 0-20% dos resíduos	Não = limpeza de 0-20% dos resíduos

As palavras grifadas em negrito nas colunas T1 e T2 referem-se às divergências entre as traduções 1 (T1) e 2 (T2)

**Legenda:T1** = Tradução 1; **T2** = Tradução 2; **T1/T2** = Versão consenso

Na etapa de retrotradução da escala BRACS, foram retraduzidos 18 segmentos da Língua Portuguesa do Brasil para a Língua Inglesa Norte-Americana, conforme apresentado na Tabela 3. Do total de segmentos, 11 foram traduzidos em concordância entre R1 e R2 e sete foram discordantes. Quanto aos segmentos discordantes, optou-se por um ajuste como a R1 e seis ajustes como a R2. As divergências foram resolvidas na versão consenso conforme apresentado na mesma tabela.

**Tabela 3:** Retrotradução entre R1 e R2, divergências e ajustes na versão consenso da escala BRACS

BRACS	R1	R2	R1/R2
1.Localização e quantidade de resíduos	*Location and quantity of residue*	*Location and* ***amount*** *of residue*	*Location and amount of residue*
2.Marque todos os locais que se aplicam	*Check all* ***sites*** *that apply*	*Check all* ***locations*** *that apply*	*Check all locations that apply*
3.e, em seguida, indique a pior pontuação obtida em cada um deles	*and then indicate the worst score obtained for each* ***of them***	*and then indicate the worst score obtained in each*	*and then indicate the worst score obtained in each*
4.Região 1	*Region 1*	*Region 1*	*Region 1*
5.Parede lateral da faringe	*Lateral wall of the pharynx*	*Lateral wall of pharynx*	*Lateral wall of the pharynx*
6.Parede posterior da faringe	*Posterior wall of the pharynx*	*Posterior wall of the pharynx*	*Posterior wall of the pharynx*
7.Base da língua	*Base of the tongue*	*Base of the tongue*	*Base of the tongue*
8.Valéculas	***Vallecula***	***Valleculae***	*Valleculae*
9.Ponta da epiglote	*Tip of the epiglottis*	*Tip of the epiglottis*	*Tip of the epiglottis*
10.Região 2	*Region 2*	*Region 2*	*Region 2*
11.Canal lateral esquerdo e recesso piriforme esquerdo	*Left lateral canal and left* ***pyriform*** *recess*	*Left lateral canal and left* ***piriform*** *recess*	*Left lateral canal and left* ***pyriform*** *recess*
12.Canal lateral direito e recesso piriforme direito	*Right lateral canal and right* ***pyriform*** *recess*	*Right lateral canal and right* ***piriform*** *recess*	*Right lateral canal and right pyriform recess*
13.Região pós-cricóide	*Postcricoid region*	*Postcricoid region*	*Postcricoid region*
14.Bolus alimentar 1	*Food bolus*	*Food Bolus*	*Food Bolus*
15.Nenhum resíduo/vestígio residual	*No residue/residual waste*	*No residue/traces*	*No residue/traces*
16.Leve	***Light***	***Mild***	*Mild*
17.Moderado	*Moderate*	*Moderate*	*Moderate*
18.Grave	*Severe*	*Severe*	*Severe*

As palavras grifadas em negrito nas colunas R1 e R2 referem-se às divergências entre as retrotraduções 1 (R1) e 2 (R2)

**Legenda:R1** = Retrotradução 1; **R2** = Retroradução 2; **R1/R2** = Versão consenso

Na etapa de retrotradução do parágrafo de instruções, observou-se os mesmos números de concordância e divergências obtidos na tradução. Apenas um segmento foi concordante entre as R1 e R2, enquanto os outros 13 foram discordantes, como pode-se observar na Tabela 4. As divergências foram resolvidas na versão consenso apresentada na mesma tabela.

**Tabela 4:** Retrotradução entre R1 e R2, divergências e ajustes na versão consenso do parágrafo de instruções da escala BRACS

PARÁGRAFO	R1	R2	R1/R2
1.A BRACS é uma escala ordinal de 11 pontos que mensura o grau de comprometimento de resíduos faríngeos	*BRACS (The Boston Residue and Clearance Scale) is na 11-point ordinal scale that measures the degree of* ***compromisse*** *of pharyngeal residue*	***The*** *BRACS (Boston Residue and Clearance Scale) is an 11-point ordinal scale that measures the degree of* ***impairment*** *of pharyngeal residue*	*The BRACS (Boston Residue and Clearance Scale) is na 11-point ordinal scale that measures the degree of impairment of pharyngeal residue*
2.A escala determina, em específico, a quantidade de resíduos faríngeos	*The scale specifically determines the amount of pharyngeal residue*	*The scale specifically determines the amount of pharyngeal residue*	*The scale specifically determines the amount of pharyngeal residue*
3.Nenhum resíduo/vestígio residual	*No residue/* ***vestigial residue***	*No residue/* ***traces***	*No residue/traces*
4.Leve = resíduos cobrindo/preenchendo <1/3 do local	***light*** *= residue covering/filling <1/3 of the* ***site***	***mild*** *= residue covering/filling <1/3 of the* ***location***	*mild = residue covering/filling <1/3 of the location*
5.Moderado = Resíduos cobrindo/preenchendo 1/3 – 2/3 do local	*moderate = residue covering/filling 1/3 – 2/3 of the* ***site***	*moderate = residue covering/filling 1/3 – 2/3 of the* ***location***	*moderate = residue covering/filling 1/3 – 2/3 of the location*
6.Grave = resíduos cobrindo/preenchendo >2/3 do local	*severe = residue covering/filling >2/3 of the* ***site***	*severe = residue covering/filling >2/3 of the* ***location***	*severe = residue covering/filling >2/3 of the location*
7.A quantidade de resíduos é pontuada considerando 12 locais da laringofaringe	*The amount of residues* ***punctuated*** *in 12* ***locations of laryngopharynx***	*The amount of residue is* ***scored*** *at 12* ***laryngopharyngeal sites***	*The amount of residue is scored at 12 laryngopharyngeal locations*
8.Se houver resíduos em quatro ou mais regiões anatômicas, adicione um ponto à somatória	***If there is residue in four or more anatomical regions, na extra point must be added to the sum***	***An extra point is added to the sum if residue is present in four or more anatomical regions***	*If there is residue in four or more anatomical regions, na extra point must be added to the sum*
9.Se houver resíduos no interior do vestíbulo, colocando o indivíduo em maior risco de aspiração após a deglutição, adicione um ponto à somatória	***If there is residue inside the vestibule, placing the individual at increased risk of aspiration after swallowing, another extra point must be added to the sum***	***Another point is added to the sum if there is residue inside the vestibule, putting the individual at greater risk of aspiration after swallowing***	*If there is residue inside the vestibule, placing the individual at increased risk of aspiration after swallowing, another extra point must be added to the sum*
10.Se houver resíduos e o indivíduo não manifestar deglutições espontâneas, adicione um ponto à somatória, considerando que, aparentemente, existe a falta de sensação faríngea	***And if there is residue and the individual does not manifest spontaneous swallows, another extra point must be added to the sum, considering that, apparently, there is a*** *lack of pharyngeal sensation*	***An additional point is added to the sum if there is residue and the individual does not manifest spontaneous swallowing, indicating an apparent*** *lack of pharyngeal sensation*	*And if there is residue and the individual does not manifest spontaneous swallows,another extra point must be added to the sum, indicating na apparent lack of pharyngeal sensation*
11.Por fim, as deglutições espontâneas ou estimuladas são, então, julgadas quanto a sua eficiência	*Finally, spontaneous or stimulated swallows are judged* ***by*** *their efficiency*	*Finally, spontaneous or stimulated swallows are judge* ***according to*** *their efficiency*	*Finally, spontaneous or stimulated swallows are judge according to their efficiency*
12.Sim = limpeza de 80-100% dos resíduos	*Yes =* ***cleaning*** *80-100% of residue*	*Yes = 80-100% of residue* ***cleared***	*Yes = 80-100% of residue cleared*
13.Parcialmente = limpeza de 20-80% dos resíduos	*Partially =* ***cleaning*** *of 20-80% of residue*	*Partially = 20-80% of residue* ***cleared***	*Partially = 20-80% of residue cleared*
14.Não = limpeza de 0-20% dos resíduos	*No =* ***clearing of*** *0-20% of residue*	*No = 0-20% of residue* ***cleared***	*No = 0-20% of residue cleared*

As palavras grifadas em negrito nas colunas R1 e R2 referem-se às divergências entre as retrotraduções 1 (R1) e 2 (R2)

**Legenda:R1** = Retrotradução 1; **R2** = Retroradução 2; **R1/R2** = Versão consenso

Após a finalização desta etapa, conforme sugestão dos tradutores e opção dos autores do estudo, foi realizada a comparação entre a escala BRACS retrotraduzida e a versão original, a fim de confirmar as equivalências. Neste processo, realizado em uma reunião por plataforma digital, foram discutidas questões semânticas e gramaticais relacionadas à anatomia das regiões citadas, onde não houve discordâncias.

Por fim, na E3, em relação aos resultados do processo de validade de conteúdo da seleção das imagens, a concordância entre os juízes foi de 0,958 com intervalo de confiança de 0,051 e 0,036. Os resultados sugerem concordância quase perfeita entre os juízes. Os juízes apresentaram três discordâncias, em ambas regiões 1 e 2, referente às localizações e grau de comprometimento, sendo elas: Parede lateral da faringe e parede posterior da faringe - Leve; Valéculas e ponta da epiglote - Moderado; Região Pós-cricóidea - Moderado.

Quanto à primeira discordância da localização, um juiz sugeriu que a imagem escolhida representava mais a localização de “parede lateral da faringe” do que ambas (“Parede lateral e parede posterior da faringe”), na segunda discordância da localização “Valéculas e ponta da epiglote” outro juiz sugeriu que para comparar os níveis houvesse mais imagens de “ponta de epiglote” classificação de gravidade (leve e grave) e, por último, outra sugestão dos juízes na “Região Pós-cricóidea” foi o comentário sobre a dificuldade em analisar o grau de comprometimento na imagem estática, gerando dúvidas entre grau “moderado” e “grave”. As discordâncias foram consideradas pelos autores, e em forma de consenso, como representativas do grau do grau de comprometimento mantendo-se as imagens selecionadas.

Por fim, as imagens em concordância para representar cada região e níveis propostos previamente na BR-BRACS estão representadas conforme mostra o Apêndice A.

## DISCUSSÃO

A escassez de instrumentos de avaliação e classificação do grau de comprometimento para disfagia orofaríngea, com alguma evidência de validade e elaborados na Língua Portuguesa do Brasil, é facilmente detectada em uma revisão simples da literatura. Desta forma, a tradução e adaptação de um instrumento disponível em outro idioma demonstra-se frequentemente como possibilidade frente a este problema, desde que realizada com metodologia adequada^(11)^. Observa-se também que o processo de tradução apresenta grande variabilidade de diretrizes e recomendações e, muitos autores acabam adaptando as etapas seguidas com a combinação de uma ou mais metodologias^(12-15)^.

No processo de tradução e retrotradução as divergências semânticas e sintáticas são frequentes e neste processo com a BR-BRACS aconteceram em maior quantidade na tradução do parágrafo de instruções do que na escala propriamente dita. Esta questão é facilmente entendida quando comparada com outras traduções que também trataram de escalas baseadas em regiões anatômicas, onde as divergências nunca estão nos próprios termos da anatomia humana^(16-18)^. Entende-se claramente que as traduções com frases mais extensas são de maior probabilidade de divergências, assim como mencionado em outros estudos de tradução, onde as dificuldades descritas pelos autores são voltadas para análise de discrepâncias semânticas, idiomáticas, conceituais, linguísticas e contextuais^(19-25)^.

Além da questão da extensão das frases e seu impacto nas traduções com divergências sintáticas, ocorreram questões semânticas relacionadas aos corretos termos usados na área da saúde. Essa questão técnica das traduções, assim como afirmaram outros estudos, ocorre frequentemente devido à adaptação transcultural, como por exemplo a tradução do termo “*cookie*” para “bolacha recheada”^(12)^ e dificuldades com itens que não apresentam tradução aproximada no idioma alvo que tenha o mesmo significado proposto pelo autor original^(20)^.

Para potencializar a discussão sobre as traduções, e no contexto da síntese das traduções com a participação dos autores responsáveis pelo estudo, poucos artigos analisados citaram a participação dos autores das escalas disponíveis para realização da síntese das traduções e análise em conjunto aos tradutores, por outro lado mencionaram a importância da formação e/ou anos de experiência na área de tradução ou atuação com a área do estudo^(24-26)^.

Os profissionais tradutores deste estudo apresentaram média de experiência acima de cinco anos na área de tradução e assim como as etapas descritas por Beaton et al.^(9)^, e uma das tradutoras responsáveis pela primeira etapa estava ciente dos conceitos técnicos presentes no instrumento selecionado, ou seja, configurando-se com *expertise* na área. Os resultados obtidos na etapa de tradução e retrotradução foram dentro do esperado, obtendo-se uma tradução que refletiu a linguagem e seus aspectos semânticos, sintáticos e gramaticais. Por fim, entendendo que somente a tradução e retrotradução de um instrumento não garante suficiente evidências de validade, ou seja, embora o modelo de tradução final tenha culminado em uma escala traduzida e retrotraduzida que possibilita aplicação clínica, é necessário a reunião de muitas outras evidências para assegurar a validade e confiabilidade do instrumento no que se propõe a mensurar^(15)^.

Além disso, por se tratar de uma escala que em sua proposição original não apresentou imagens representativas para possibilitar o treinamento dos profissionais que aplicam, e para prosseguir com a investigação das evidências de validade para esta escala, verificar a evidência de validade de conteúdo para a seleção e submissão de imagens era essencial para permitir o uso futuro de forma confiável explore pesquisador e pelo clínico.

As discordâncias citadas nos resultados abordaram diferentes fatores que dificultaram a seleção de imagens para compor o instrumento traduzido. Observou-se maior dificuldade em regiões que incluem mais de uma localização como “Parede lateral e parede posterior da faringe” e “Valéculas e ponta de epiglote”, pois em diversos exames analisados não haviam ambas estruturas com presença de resíduos. Outra questão levantada pelos juízes foi a análise de imagens estáticas, como no caso da imagem da “Região pós-cricóidea”. De acordo com um dos juízes, a imagem seria de difícil classificação por possível interferência da região da parede posterior de faringe, gerando dúvida entre a classificação entre os graus “moderado” e “grave”.

Assim, embora a BRACS seja uma ferramenta que fornece a avaliação dos resíduos faríngeos em diferentes áreas da faringe, além de incluir a eficiência do mecanismo de “*clearance*”, ou seja, a limpeza dos resíduos, que a difere de outras escalas^(6,8)^, a possibilidade de marcadores claros sobre os níveis de resíduos faríngeos tem se constituído como um forte instrumento para auxiliar, perante demais achados clínicos e instrumentais como um dos aspectos relevantes na tomada de decisão clínica em disfagia orofaríngea.

Por outro lado, destaca-se que a ausência de imagens na escala BRACS original, dificulta a exata compreensão da expectativa dos autores originais frente aos elementos visuais necessários para o treinamento e aplicabilidade da escala, o que nos permitiu avançar e propor imagens que possam facilitar esse processo.

No contexto da representação visual por meio da escolha de imagens estáticas na representação de achados da deglutição no contexto da classificação do grau de comprometimento, essa tem sido uma opção frequente em outras escalas de resíduos faríngeos^(6.8)^. Embora as imagens sejam estáticas, ao se tratar de resíduos faríngeos há a questão que envolve o volume e os marcadores anatômicos das regiões envolvidas permitindo a diferenciação entre os níveis. No entanto, cabe ressaltar que para todo método de análise qualitativa e quantitativa em deglutição, sempre será necessário um processo de treinamento do avaliador visando minimizar a subjetividade da análise e manter no processo a questão da confiabilidade do instrumento.

Assim, finalizada essa etapa do processo, e certos da necessidade em prosseguir para confirmar as vantagens e desvantagens desse instrumento para mensurar RF na VED, ressalta-se que serão dados novos passos em direção a futuras contribuições a este método diagnóstico de classificação dos RF em disfagia orofaríngea.

## CONCLUSÃO

O processo de tradução e adaptação transcultural foi realizado e a seleção das imagens para análise perceptivo visual de resíduos faríngeos da BR*-*BRACS foi concluída com evidência de validade de conteúdo.

## Figures and Tables

**Figure:** Parede lateral da faringe e parede posterior da faringe

**Figure:** Base de língua

**Figure:** Valéculas, ponta da epiglote

**Figure:** Canal lateral e recesso piriforme esquerdo e/ou direito

**Figure:** Região pós-cricóidea

## Apêndice A Modelo da estruturação da escala BR-BRACS com as imagens selecionadas

A BRACS (The Boston Residue and Clearance Scale) é uma escala ordinal de 11 pontos que mensura o grau de comprometimento dos resíduos faríngeos. A escala determina, especificamente, a quantidade de resíduos faríngeos (nenhum resíduo/vestígio residual; leve = resíduos cobrindo/preenchendo <1/3 do local; moderado = resíduos cobrindo/preenchendo 1/3 - 2/3 do local; grave = resíduos cobrindo/preenchendo >2/3 do local). A quantidade de resíduos é pontuada em 12 locais da laringofaringe.

Se houver resíduos em quatro ou mais regiões anatômicas, um ponto extra deve ser adicionado à somatória. Se houver resíduos no interior do vestíbulo, colocando o indivíduo em maior risco de aspiração após a deglutição, outro ponto extra deve ser adicionado à somatória. E, se houver resíduos e o indivíduo não manifestar deglutições espontâneas, outro ponto extra deve ser adicionado à somatória, considerando que, aparentemente, há falta de sensação faríngea.

Por fim, as deglutições espontâneas ou estimuladas são julgadas quanto a sua eficiência (sim = limpeza de 80 - 100% dos resíduos; parcialmente = limpeza de 20 - 80% dos resíduos; não = limpeza de 0 - 20% dos resíduos).

**Table:**

Localização e quantidade de resíduos – Marque todos os locais que se aplicam e, em seguida, indique a pior pontuação obtida em cada um deles.	Bolus alimentar
	Nenhum resíduo/vestígio residual Leve Moderado Grave
**Região 1**	
Parede lateral da faringe, parede posterior da faringe	
Base da língua	
Valéculas, ponta da epiglote	
**Região 2**	
Canal lateral esquerdo e recesso piriforme esquerdo	
Canal lateral direito e recesso piriforme direito	
Região pós-cricóidea
